# Identification of potential hub genes linked to immune and metabolic alterations in postoperative systemic inflammatory dysregulation

**DOI:** 10.3389/fimmu.2023.1238774

**Published:** 2023-09-08

**Authors:** Silu Cao, Jinxuan Tang, Miaomiao Fei, Qi Jing, Fanbing Meng, Meixian Zhang, Qidong Liu, Hui Zhang, Cheng Li

**Affiliations:** ^1^ Department of Anesthesiology and Perioperative medicine, Shanghai Key Laboratory of Anesthesiology and Brain Functional Modulation, Clinical Research Center for Anesthesiology and Perioperative Medicine, Translational Research Institute of Brain and Brain-Like Intelligence, Shanghai Fourth People's Hospital, School of Medicine, Tongji University, Shanghai, China; ^2^ Key Laboratory of Spine and Spinal Cord Injury Repair and Regeneration of Ministry of Education, Orthopedic Department of Tongji Hospital, Tongji University School of Medicine, Shanghai, China

**Keywords:** postoperative systemic inflammatory dysregulation, hub genes, metabolism, C-reactive protein, surgery

## Abstract

**Background:**

Postoperative systemic inflammatory dysregulation (PSID) is characterised by strongly interlinked immune and metabolic abnormalities. However, the hub genes responsible for the interconnections between these two systemic alterations remain to be identified.

**Methods:**

We analysed differentially expressed genes (DEGs) of individual peripheral blood nucleated cells in patients with PSID (n = 21, CRP > 250 mg/L) and control patients (n = 25, CRP < 75 mg/L) following major abdominal surgery, along with their biological functions. Correlation analyses were conducted to explore the interconnections of immune-related DEGs (irDEGs) and metabolism-related DEGs (mrDEGs). Two methods were used to screen hub genes for irDEGs and mrDEGs: we screened for hub genes among DEGs via 12 algorithms using CytoHubba in Cytoscape, and also screened for hub immune-related and metabolic-related genes using weighted gene co-expression network analysis. The hub genes selected were involved in the interaction between changes in immunity and metabolism in PSID. Finally, we validated our results in mice with PSID to confirm the findings.

**Results:**

We identified 512 upregulated and 254 downregulated DEGs in patients with PSID compared with controls. Gene enrichment analysis revealed that DEGs were significantly associated with immune- and metabolism-related biological processes and pathways. Correlation analyses revealed a close association between irDEGs and mrDEGs. Fourteen unique hub genes were identified via 12 screening algorithms using CytoHubba in Cytoscape and via weighted gene co-expression network analysis. Among these, *CD28*, *CD40LG*, *MAPK14*, and *S100A12* were identified as hub genes among both immune- and metabolism-related genes; these genes play a critical role in the interaction between alterations in immunity and metabolism in PSID. The experimental results also showed that the expression of these genes was significantly altered in PSID mice.

**Conclusion:**

This study identified hub genes associated with immune and metabolic alterations in patients with PSID and hub genes that link these alterations. These findings provide novel insights into the mechanisms underlying immune and metabolic interactions and new targets for clinical treatment can be proposed on this basis.

## 1 Introduction

Postoperative systemic inflammatory dysregulation (PSID), a state of inflammation that may occur postoperatively, can be identified through postoperative phenotypic changes, such as elevated levels of C-reactive protein (CRP) and pro-inflammatory cytokines. PSID is an important feature of postoperative sepsis (1, 2). The Third International Consensus Definition for Sepsis indicates that underlying inflammation and metabolic abnormalities substantially increase the risk of mortality (3).

During surgical procedures, the body is exposed to innate pathogens and cellular debris that can activate the immune system (4), while severe tissue damage can result in higher levels of inflammatory mediators and cytokine release, ultimately driving immune, metabolic, and hormonal processes and leading to a stress response. Although the inflammatory immune response is essential for repairing damage and fighting harmful products, it can lead to PSID, which increases the risk of complications, prolongs hospital stays, and may cause death. PSID increases the risk of postoperative infection and induces inflammation-mediated complications and organ dysfunction (4–8). Metabolic alterations, including changes in energy and nitrogen balance, as well as the utilisation of substrates such as proteins, carbohydrates, and lipids, influence the occurrence of postoperative complications (9). These processes can alter glucose and protein catabolism and can cause hormonal dysregulation and other effects that impede recovery and increase morbidity (10).

The transition of immune responses from quiescent to activated states involves multiple metabolic pathways (11). Both innate and adaptive immune cells increase their metabolic flux upon stimulation, promoting energy production and biosynthesis while restoring metabolic pathways to support proliferation, effector molecule production, and cell differentiation (12–15). However, few studies have focused on the correlation between immunological and metabolic changes in patients following surgery, and even fewer have prospectively observed the early postoperative period before clinical signs become evident. CRP, an acute-phase protein, has a half-life of 19 h, and white cell count is a commonly used marker of postoperative inflammation and infection. Investigation and identification of the potential hub genes associated with immune and metabolic alterations in patients with PSID and linking these alterations should provide insights into the mechanisms underlying immune and metabolic interactions and new targets for clinical treatment (16).

**Figure 1:**
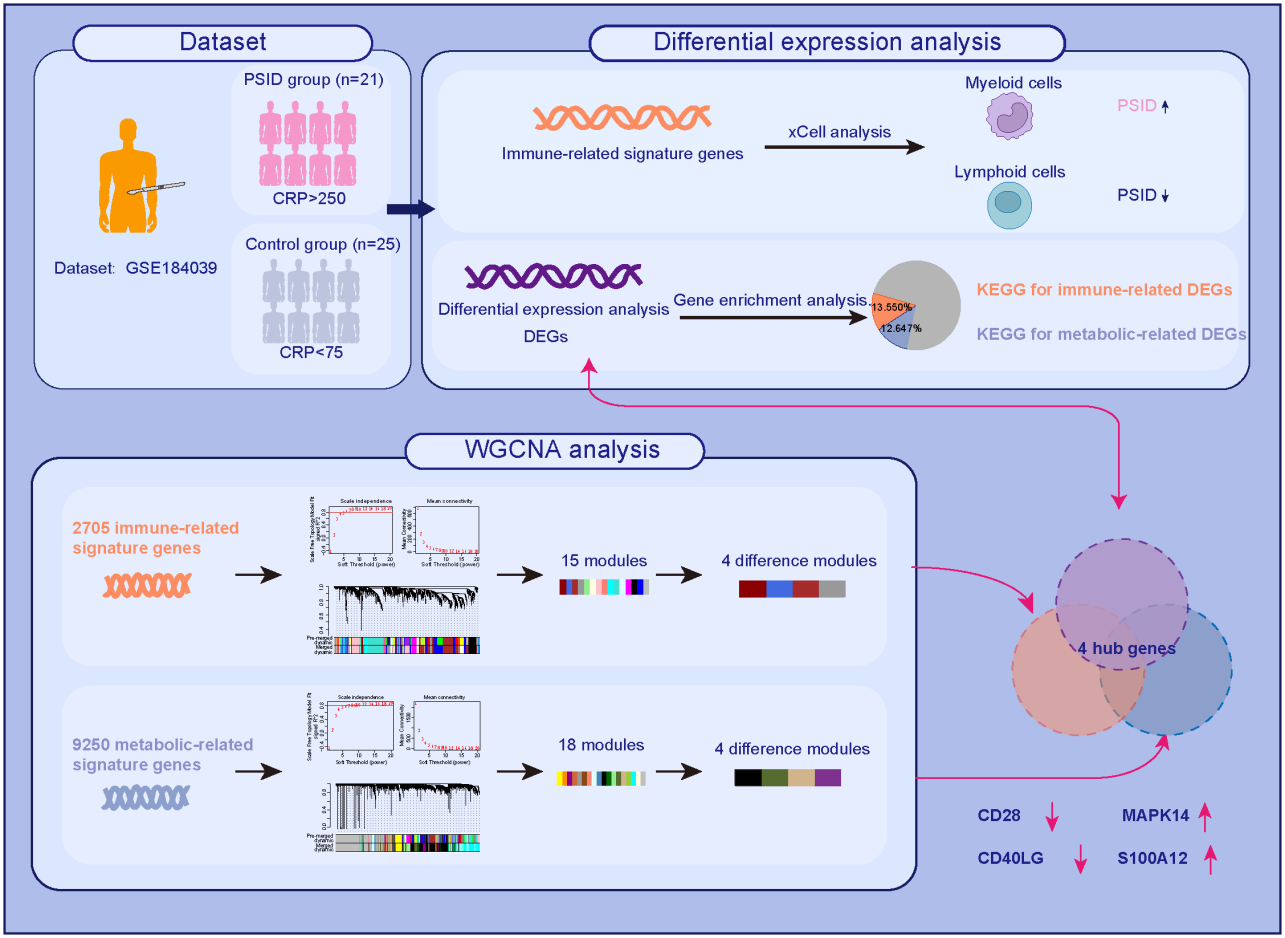
The workflow of this study.

In this study, we comprehensively analysed the public RNA-seq dataset GSE184039 to evaluate differential genetic characteristics and alterations to biological processes in patients with high and low levels of CPR after major abdominal surgery. We screened hub genes associated with both immune and metabolic changes in patients with PSID. Our results provide a new perspective on the diagnosis and treatment of PSID. The workflow of this study is illustrated in **Figure 1**.

## 2 Results

### 2.1 DEGs in PSID and function enrichment analysis

**Figure 2:**
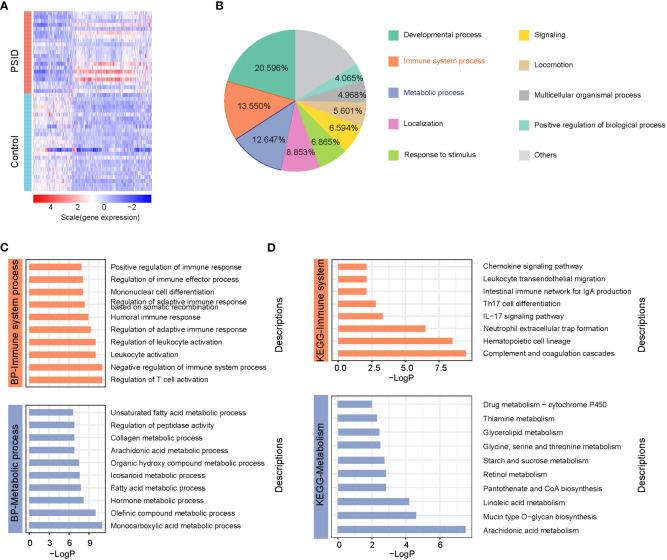
Widespread alterations to immune system and metabolic processes are associated with PSID. **(A)** Heatmap of 766 differentially expressed genes between the PISD and Control groups. DEGs were filtered on the criteria |log2 fold change (FC)|≥1 and adjusted p value < 0.05. **(B)** Biological functions of the biological processes associated with 766 DEGs, annotated based on Gene Ontology (GO) enrichment analysis. **(C)** Top 10 biological processes associated with immunology and metabolism enriched by 766 DEGs based on GO enrichment analysis. **(D)** 8 immune-related pathways and 10 metabolism-related pathways were significantly enriched by 766 DEGs based on KEGG enrichment analysis.

Significant alterations associated with immunity and metabolism were detected in patients with PSID. A total of 766 differentially expressed genes (DEGs), with absolute log2(fold change) ≥ 1 and adjusted P value < 0.05, were identified (512 upregulated and 254 downregulated in the PSID group). A heatmap of DEGs in the high- and low-CRP groups is presented in **Figure 2A**. In terms of biological process functions, the DEGs were significantly enriched in immune system processes (13.550%) and metabolic processes (12.647%) (**Figure 2B**). The top 10 biological processes related to the immune system process and the top 10 biological processes related to the metabolic process are shown in **Figure 2C**. These findings suggest significant differences between the groups in terms of the immune and metabolic processes involved in PSID. We performed Kyoto Encyclopedia of Genes and Genomes (KEGG) pathway enrichment analyses to investigate the potential pathways involved in immune and metabolic changes in PSID and found that 8 immune-related pathways and 10 metabolism-related pathways were significantly enriched (**Figure 2D**). The most prominent of these were the complement and coagulation cascades and arachidonic acid metabolism. The enriched DEGs for specific immune- and metabolic-related KEGG pathways were plotted using network plots (**Supplementary Figures 2A, B**). Most DEGs were enriched in complement and coagulation cascades; theIL-7 signalling pathway; leukocyte transendothelial migration; neutrophil extracellular trap formation; arachidonic acid metabolism; glycerolipid metabolism; glycine, serine, and threonine metabolism; pantothenate and CoA biosynthesis; starch and sucrose metabolism. Additionally, 88 genes were identified in screening based on the top 20 genes selected according to the 12 different analysis methods via the CytoHubba tool, of which 31 genes detected using at least three different methods were considered to be hub genes among the DEGs (**Table 1**).

**Table 1:** Number of identifications (*n*) of the top 20 hub genes selected using 12 algorithms via the CytoHubba tool in Cytoscape.

Gene	*n*	Genes	*n*	Gene	*n*	Gene	*n*	Gene	*n*
ALB	10	CD28	4	FLT3LG	2	CYP4F3	1	MAGED4	1
IL6	10	CEBPB	4	HIST1H2AI	2	F12	1	MAGED4B	1
PPARG	10	CTGF	4	HIST1H2BC	2	FBLN5	1	MAL	1
IL10	9	CXCL9	4	HIST2H2AB	2	FFAR3	1	MS4A4A	1
ITGAM	9	IL7R	4	NR3C2	2	HBM	1	OSM	1
MMP9	9	ALOX5	3	PRL	2	HIST1H1C	1	PLIN5	1
CCL2	8	ARG1	3	SOCS3	2	HIST1H1E	1	RHOU	1
MMP2	8	COL5A1	3	SYN1	2	HIST1H3J	1	SFTPD	1
BMP4	7	GPR29	3	CCL23	1	HIST1H4F	1	SLC22A31	1
CD163	6	KCNH7	3	CD207	1	HIST2H2AA	1	SLC39A8	1
MAPK14	6	LEF1	3	CD27	1	HIST2H3D	1	STX3	1
NT5E	6	NES	3	CEACAM3	1	HLF	1	TREML4	1
S100A12	6	SCN5A	3	CEP55	1	IL1R2	1	TRIB2	1
CCR7	5	ACE	2	CLDN9	1	IL22	1	TROAP	1
CD40LG	5	CD1B	2	COL8A2	1	IL23R	1		
HGF	5	CD1E	2	CTSD	1	IRAK3	1		
RETN	5	CD276	2	CYP2C9	1	LPL	1		
CD1C	4	CSF3R	2	CYP4F2	1	LRG1	1		

### 2.2 Analyses of immune scores

**Figure 3:**
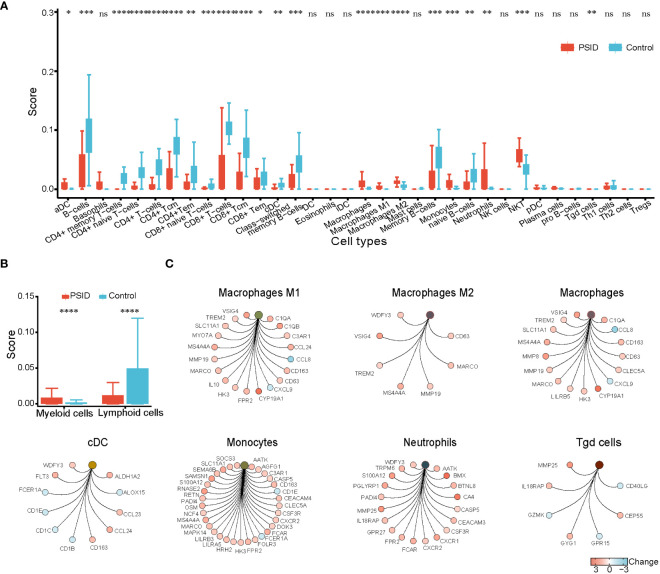
Enrichment of immune cells. **(A)** Differences in immune cell scores between the PSID group (n=21) and Control group (n=25) (p < 0.05). Asterisks indicate significant difference between the two groups: *P < 0.05, **P < 0.01, ***P < 0.001, ****P < 0.0001. **(B)** Differences in myeloid cells and lymphoid cell scores between the PSID group (n=21) and Control group (n=25). Asterisks indicate significant differences between the two groups: ****P < 0.0001. **(C)** Specific genes associated with these myeloid cells, based on cnetplot analysis. ns, P>0.05.

The immune scores of various cell types were determined using the xCell package according to the expression profile of immune cell signature genes; immune scores for 34 immune cells are shown in **Figure 3A**. In a comparison between the high- and low-CRP groups in terms of immune cell scores, B cells, CD4+ memory T-cells, TD4+ naïve T-cells, CD4+ T-cells, CD4+Tcm, CD4+Tem, TD8+ naïve T-cells, CD8+ T-cells, CD8+Tcm, CD8+Tem, cDC, class-switched memory B-cells, and naïve B-cells had lower scores in the PSID group (P < 0.05). In contrast, macrophages, macrophages M1, macrophages M2, monocytes, neutrophils, NKT cells, and Tg cells had higher scores. In a comparison between the groups in terms of immune cell scores for myeloid and lymphoid cells, scores for myeloid cells were elevated in the PSID group (P < 0.05); in contrast, lymphoid cells had lower scores (**Figure 3B**). The enriched DEGs for specific immune cells were plotted using net plots (**Figure 2C** and **Supplementary Figure 3**). Most DEGs enriched in macrophages, monocytes, and neutrophils were expressed more in the PSID group than in the Control group (**Figure 3C**), while most DEGs enriched in lymphoid cells were expressed less in the PSID group (**Supplementary Figure 3A**).

### 2.3 Correlation between immunity and metabolism in PSID

**Figure 4:**
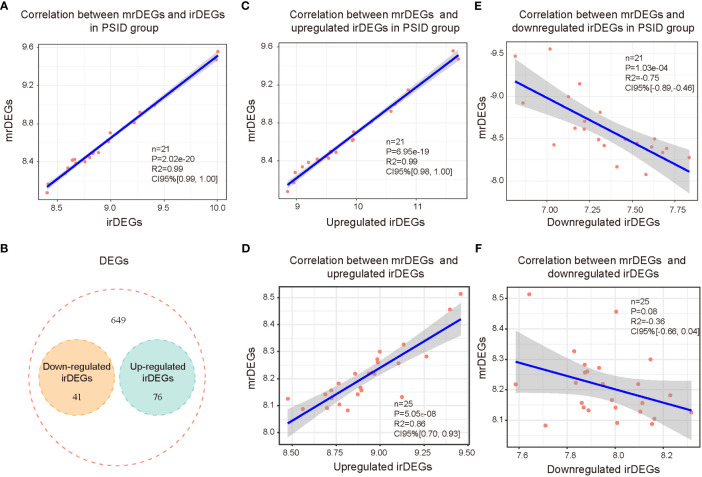
Correlation between immunity and metabolism in PSID. **(A)** Correlation analysis of all immune-related genes and metabolism-related genes after surgery. **(B)** Venn diagram displaying the intersections of the 766 DEGs with upregulated and downregulated irDEGs. **(C)** Correlation analysis of postoperatively upregulated irDEGs and mrDEGs in the PSID group. **(D)** Correlation analysis of postoperatively upregulated irDEGs and mrDEGs in the Control group. **(E)** Correlation analysis of mrDEGs and postoperatively downregulated irDEGs in the PSID group. **(F)** Correlation analysis of mrDEGs and postoperatively downregulated irDEGs in the Control group.

To elucidate the relevance of immune and metabolic alterations in patients with PSID, we first analysed the correlations between irDEGs and mrDEGs. The results showed a significant positive correlation between irDEGs and mrDEGs in the PSID group (R^2 =^ 0.99, **Figure 4A**). To further examine this correlation, we further analysed 41 downregulated and 76 upregulated immune-related genes (**Figure 4B**) among the 766 DEGs. The results showed that upregulated irDEGs were positively correlated with mrDEGs (R^2 =^ 0.99, **Figure 4C**) in the PSID group. This correlation was weaker in the Control group (R^2 =^ 0.86, **Figure 4D**). Analysis of downregulated the irDEGs also showed that the immune–metabolic correlation was stronger in the PSID group (R^2^ = -0.75, **Figure 4E**) than in the Control group (R^2^ = -0.36, **Figure 4F**). These results suggest a close correlation between immunity and metabolism in patients with PSID.

### 2.4 Hub genes involved in immune-related genes

**Figure 5:**
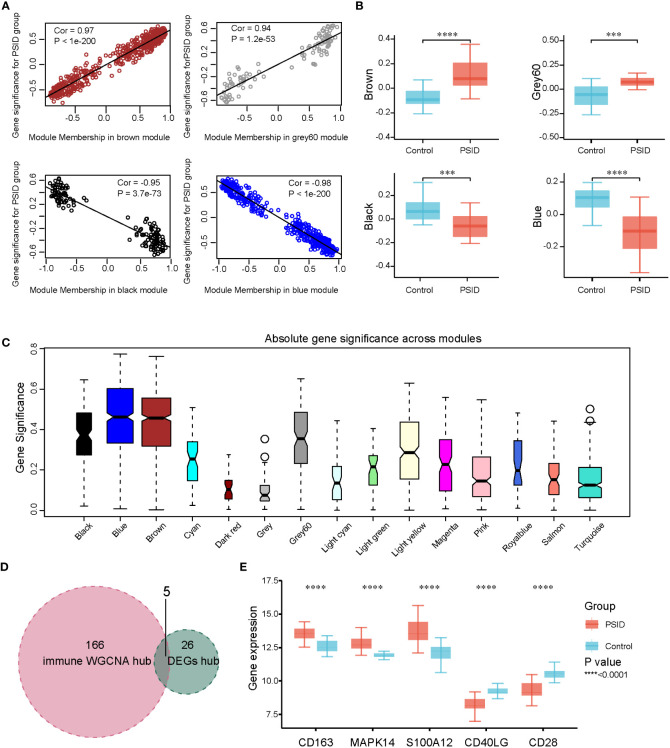
WGCNA and identification of significant modules among immune-related genes. **(A)** Scatterplots of gene significance against MM in the brown, grey60, black, and blue modules. **(B)** Comparison of immune cell signature genes in the four modules between the PSID group (n=21) and the Control group (n=25). Asterisks indicate significant difference between the two groups: ***P < 0.001, ****P < 0.0001. **(C)** Gene significance across 15 modules. **(D)** Venn diagram illustrating the intersection between the 171 immune hub genes identified using WGCNA method and the 31 immune hub genes based on cell signatures. The 5 genes at the intersection of these two groups are shown in panel **(E)**. **(E)** Differential expression analysis of five key genes for the PSID and Control groups. Asterisks indicate significant differences between the two groups: ****P < 0.0001.

To identify the hub genes associated with immune changes in PSID, we performed weighted gene co-expression network analysis (WGCNA) for 2,705 immune cell signature genes to construct gene expression networks. Samples were excluded based on standardised connectivity values < -5, and all 46 samples were included in the WGCNA. Furthermore, a soft-threshold value (β) of 7 is considered to be the optimal soft-threshold parameter for construction of a gene expression network. Using this parameter, we obtained 21 gene expression modules for immune cell signature genes (**Supplementary Figures 4A, B**). The modules were merged according to a correlation coefficient of > 0.75, resulting in 15 modules. The correspondences between the 15 modules and age, sex, and CRP group were identified to detect the correlations between them (**Supplementary Figure 4C**). Using the criteria of absolute value of the correlation coefficient > 0.5 and P < 0.05, the brown, grey60, black, and blue modules were found to be closely associated with the high-CRP group, and gene significance was closely associated with module membership (MM) in these modules (**Figure 5A**). There were significant differences in gene expression between the high- and low-expression groups in these four modules (**Figure 5B**). Absolute gene significance in the modules is shown in **Figure 5C**. A total of 171 hub genes among the immune cell signature genes were identified through screening with the criteria of absolute gene significance > 0.6 and MM > 0.8. Finally, five intersecting genes (*CD163*, *MAPK14*, *S100A12*, *CD40LG*, and CD28) were obtained after merging hub genes among the DEGs and hub genes among immune cell signature genes (**Figure 5D**). These were identified as immune-related hub genes for PSID. Compared with the low-CRP group, *CD163*, *MAPK14*, and *S100A12* were increased, while *CD40LG* and *CD28* were decreased in the PSID group (**Figure 5E**).

### 2.5 Hub genes involved in metabolism-related genes

**Figure 6:**
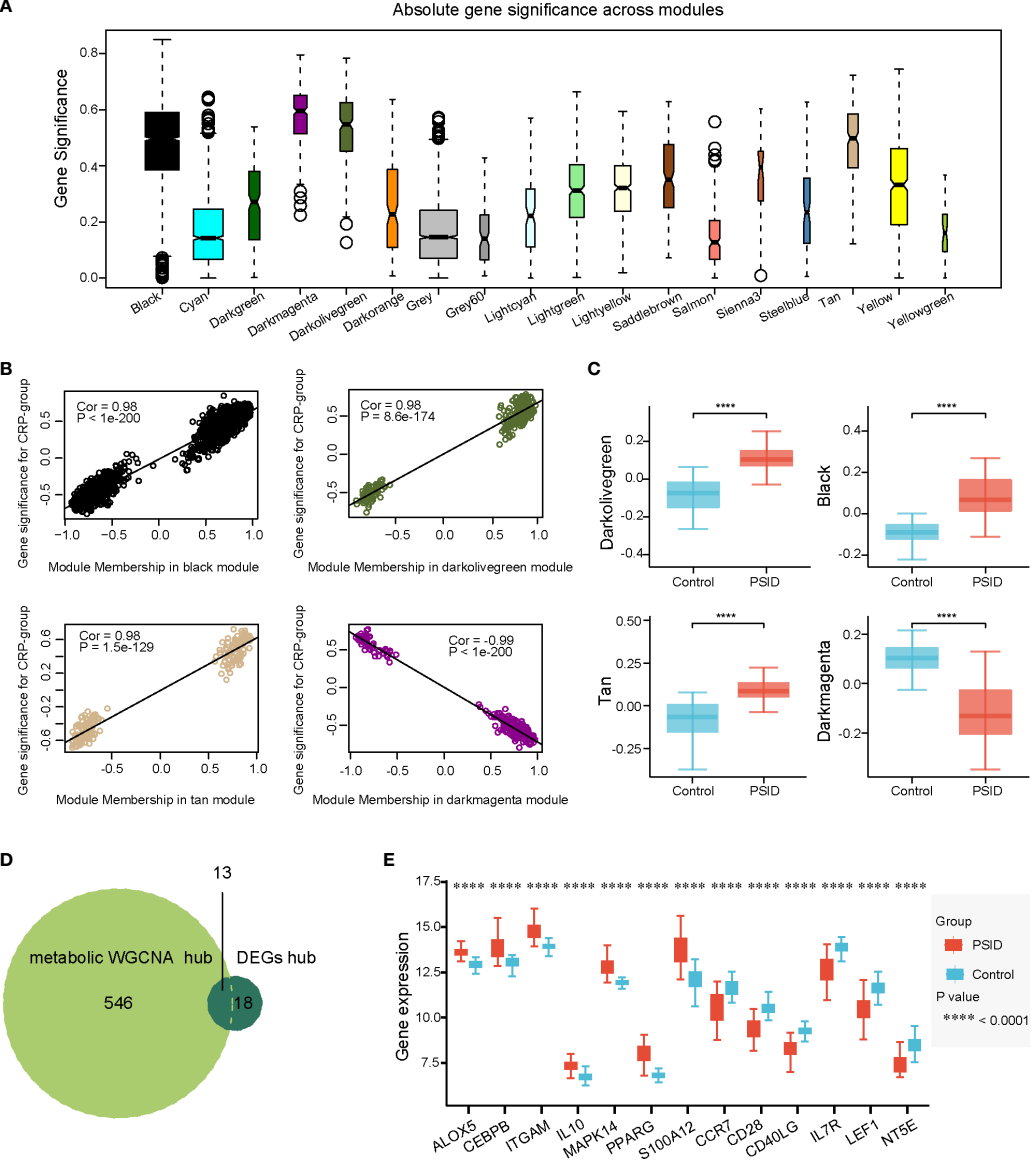
Selection of metabolic-related hub genes. **(A)** Gene significance across 18 modules. **(B)** Scatterplots of GS against MM in the dark olive green, tan, black, and dark magenta modules. **(C)** Comparison of metabolism-related genes in the four modules between the PSID group (n=21) and the Control group (n=25) (p < 0.05). Asterisks indicate significant differences between the two groups: ****P < 0.0001. **(D)** Venn diagram illustrating the intersection between the 559 metabolic hub genes identified using the WGCNA method and the 31 immune hub genes based on cell signatures. The 13 genes at the intersection of these two groups are shown in panel **(E)**. **(E)** Differential expression analysis of 13 key genes for the PSID and Control groups. Asterisks indicate significant difference between the two groups: ****P < 0.0001.

The same method was used to identify hub genes associated with metabolic changes in PSID. We used *β* = 17 to construct gene expression networks (**Supplementary Figure 5A**). Ultimately, 37 gene co-expression modules were identified (**Supplementary Figure 5B**). The modules were merged according to a correlation coefficient of > 0.75, resulting in 18 modules. The correspondences of the 18 modules with age, sex, and CRP group were also identified in order to detect correlations between them (**Supplementary Figure 5C**). The dark olive green, tan, black, and dark magenta modules were associated with the PSID group, and gene significance was closely associated with MM in these modules (**Figure 6A**). There were significant differences in gene expression between the high- and low-expression groups in these four modules (**Figure 6B**). Absolute gene significance in the modules is shown in **Figure 6C**. A total of 559 hub genes among the metabolism-related genes were identified through screening with the criteria of absolute gene significance > 0.6 and MM > 0.8. Finally, 13 intersecting genes, namely *ALOX5*, *CEBPB*, *ITGAM*, *IL10*, *MAPK14*, *PPARG*, *S100A12*, *CCR7*, *CD28*, *CD40LG*, *IL7R*, *LEF1*, and *NT5E*, were obtained after merging hub genes among the DEGs and hub genes among the metabolism-related genes (**Figure 6D, E**). Compared with the Control, *ALOX5*, *CEBPB*, *ITGAM*, *IL10*, *MAPK14*, *PPARG*, *S100A12*, *CCR7*, *CD28*, and *CD40LG* were increased in the PSID group, while *L7R*, *LEF1*, and *NT5E* were decreased (**Figure 5E**).

### 2.6 Hub genes linked with immune and metabolism alterations in PSID

**Figure 7:**
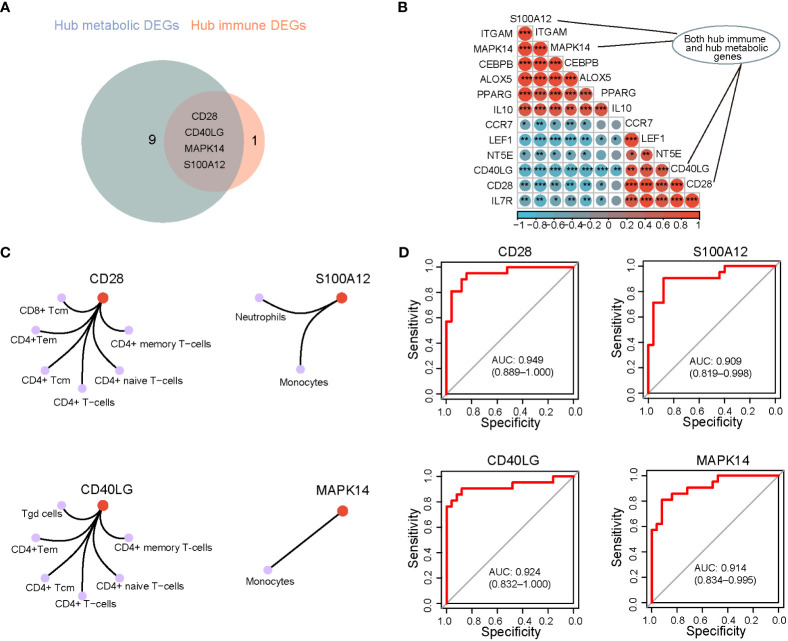
Identification of the key genes related to PSID. **(A)** Venn diagram illustrating the number of hub metabolic and hub immune genes. **(B)** Correlation analysis of metabolic-related hub genes and PSID hub genes. **(C)** The 4 hub genes associated with specific types of immune cell, based on cnetplot analysis. **(D)** Diagnostic value of hub genes. The higher the AUC, the greater the diagnostic value of the gene. *P < 0.05, **P < 0.01, ***P < 0.001.

Fourteen unique hub genes were associated with immune and metabolic alterations in PSID. Four genes, namely *CD28*, *CD40LG*, *MAPK14*, and *S100A12*, fell into the area of overlap between hub genes among the immune cell signature genes and metabolic-related genes, defined as a gene set; these were extremely closely associated with linking of the immune and metabolic changes in PSID (**Figure 7A**). The close correlations between these four hub genes and the metabolic hub genes is shown in a heatmap in **Figure 7B**. *CD28* is a signature gene of CD8+ Tcm, CD4+ Tem, CD4+ Tcm, CD4+ T cells, naïve CD4 + T cells, and CD4+ memory T cells. S100A12 is a signature gene in neutrophils and monocytes. CD40LG is a signature gene of Tgd, CD4+ Tem, CD4+ Tcm, CD4+ T cells, CD4+ naïve T cells, and CD4+ memory T cells. Finally, MAPK14 was identified as the signature gene for monocytes (**Figure 7C**). This suggests that alterations in these cells are involved in the metabolic alterations occurring in PSID. Receiver Operating Characteristic (ROC) curves were used to validate the diagnostic value of the four hub genes in our cohort; *CD28*, *CD40LG*, *MAPK14*, and *S100A12* all had high diagnostic value, with area under the ROC curve (AUC) > 0.9 in all cases (**Figure 7D**).

### 2.7 Decreased CD28 and CD40LG expression in T cells of PSID mice

**Figure 8:**
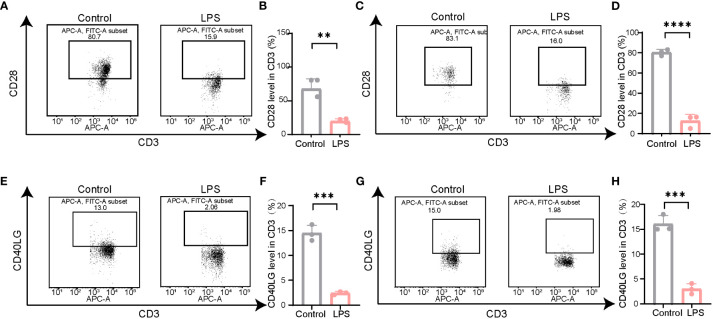
The expression of *CD28* and *CD40LG* decreased in LPS-induced mouse model. **(A)** Representative illustrations of flow cytometry gating strategy for identification of the level of *CD28* in the spleen in mice. **(B)** Statistical comparison of CD28 levels in the spleen between LPS and Control. For each group, n = 3. **P =0.0031, t = 6.384. **(C)** Representative illustrations of flow cytometry gating strategy for identification of the level of *CD28* in the blood in mice. **(D)** Statistical comparison of CD28 levels in the blood between LPS and Control. For each group, n = 3. ****P < 0.0001, t = 16.42. **(E)** Representative illustrations of flow cytometry gating strategy for identification of the level of *CD40LG* in the spleen in mice. **(F)** Statistical comparison of *CD40LG* levels in the spleen between LPS and Control. For each group, n = 3. ***P =0.0002, t = 12.65. **(G)** Representative illustrations of flow cytometry gating strategy for identification of the level of *CD40LG* in the blood in mice. **(H)** Statistical comparison of CD40LG levels in the blood between LPS and Control. For each group, n = 3. ***P =0.0004, t = 11.25. Statistical results: two-sided unpaired t-test. Values plotted are means ± SEM.

Most PBMCs are lymphocytes, including B and T cells, among which CD3^+^T cells account for the majority. According to the results of the bioinformatics analysis, the expression of *CD28* and *CD40LG* in patients with PSID was significantly reduced. To further validate the results of the biological analysis, we examined the expression of *CD28* and *CD40LG* in modulating LPS-induced PSID. Flow cytometry was used to detect the expression of *CD28* and *CD40LG* in the CD3^+^T cells of septic mice; this showed that *CD28* expression in the CD3^+^T cells of the blood (**Figures 8A, B**) and spleen (**Figures 8C, D**) was significantly decreased. The results on *CD40LG* in blood (**Figures 8E, F**) and spleen (**Figures 8G, H**) were consistent with the results of the bioinformatics analysis. These data confirm the accuracy of our bioinformatics analysis and will help to facilitate subsequent research.

## 3 Methods

### 3.1 Data collection and processing

We downloaded the gene expression matrix data for GSE184039 from the Gene Expression Omnibus (GEO) database (https://www.ncbi.nlm.nih.gov/geo/query/acc.cgi?acc=GSE184039). The GEO database is an international public repository that archives and freely distributes high-throughput gene expression datasets and other functional genomics datasets (17). This mainly consists of gene sequencing data, including microarray and second- and third-generation sequencing data, which can be downloaded using the GEO query package in the R programming environment (version 4.2.2) (18). The dataset analysed in this study contains transcriptomic data from patients undergoing major abdominal surgery. Patients were divided into a PSID group (n = 21, CRP > 250 mg/L) and a Control group (n = 25, CRP < 75 mg/L) based on postoperative levels; clinical information on all patients is listed in **Table 2**. The GSE184039 matrix contains expression data for 60,662 genes. If there were two or more expression counts for the same gene name, the average value was determined. Ultimately, 60,583 non-duplicate gene expression counts were obtained for analysis of DEGs.

**Table 2:** Characteristics of the patients who provided high-CRP and low-CRP postoperative samples in the GSE184039 dataset.

Characteristics	Control group (n = 25)	PSID (n = 21)
Age	55.88 ± 14.15	70.14 ± 10.49
Gender (male/female)	6/19	5/16
CPR (highest level)	48.48 ± 14.31	308.13 ± 54.09

### 3.2 Function enrichment analysis of DEGs and identification of hub genes

Raw counts were normalised to the vst function using the DESeq2 package. DEGs between the PSID and Control groups were screened for based on cutoff criteria of absolute log2(fold change) ≥ 1 and adjusted P value < 0.05. Biological process (BP) and (KEGG) analyses for DEGs were performed using Metascape (https://metascape.org/gp/index.html) to predict the biological functions and pathways in which the DEGs were involved (19). The threshold for statistical significance was set at P < 0.05. The biological processes and pathways associated with immunity and metabolism were detected, and data on genes enriched in the immune- and metabolism-related pathways were visualised using the igraph, ggraph, and tidygraph packages.

Potential interactions between the DEGs were analysed using the STRING database. The network with a confidence score ≥ 0.4 in STRING was retained and then input to Cytoscape (version 3.7.1) for visualisation. The top 20 genes were selected via 12 different analysis methods using the CytoHubba tool; genes detected using at least 3 different methods were considered to be hub genes among the DEGs.

### 3.3 Comparison of immune scores between two groups

The xCell tool, which uses a set of 10,808 genes to calculate the scores of 64 immune and stromal cell types based on a novel gene signature-based method, was used to calculate scores for immune cell infiltration in the peripheral blood of each sample. According to cell gene markers, 34 immune cell types were scored using xCell. A total of 21 of the immune cells were identified as lymphoid cells. Differences in cell type scores between the PSID and Control groups were estimated using the Mann–Whitney *U* test, with a threshold for statistical significance of P < 0.05.

### 3.4 Hub genes among immune-related and metabolism-related genes

WGCNA is an algorithm used to identify co-expressed gene modules with high biological significance and explore relationships between gene networks and diseases. It can also be used as a data-exploratory tool or screening method to identify key gene modules using unsupervised clustering without a priori–defined gene sets. In our study, 2,705 immune-related genes (based on immune cell signatures) were used to explore the hub genes associated with immune alterations in PSID, and 9,250 metabolism-related genes (according to the MSigDB database, https://www.gsea-msigdb.org/gsea/msigdb/) were used to explore the hub genes associated with metabolic changes. Hub genes were screened using the WGCNA package with the following steps. First, the cutreeDynamic function was used for tree pruning of the gene hierarchical clustering dendrograms, resulting in co-expression modules; correlated modules (r > 0.75) were then merged. The dissimilarity of module eigengenes was calculated using the module eigengene function. The associations between eigengene values and clinical traits were subsequently assessed using Pearson’s correlation. Hub genes were screened using criteria of gene significance > 0.6 and MM > 0.8.

### 3.5 Hub genes linked with immune and metabolism alterations in PSID

Hub genes associated with immune changes in PSID were detected by merging the hub genes with DEGs and immune-related genes. Similarly, hub genes associated with metabolic changes in PSID were detected by merging hub genes among DEGs with metabolism-related genes. Hub genes detected in relation to both immune and metabolic changes were identified as playing a critical role in linking immune and metabolic alterations in PSID. We then explored the interactions between hub genes using the corrplot package. ROC curves and their AUC were examined using the pROC package to determine the predictive value of hub genes linked to immune and metabolic alterations in PSID.

### 3.6 LPS model and flow cytometry

C57BL/6 mice were challenged via intraperitoneal injection with 10 mg/kg LPS (Sigma, Germany, L2630) or vehicle. Blood samples and spleen were collected 4 h after LPS injection. The spleen mucosa was filtered to prepare a 1ml single-cell suspension, after which 100μl peripheral blood and the spleen suspension were treated with red cell lysis solution (Biosharp, China). T cells were identified by labelling with anti-CD3-APC. For CD28 and CD40L expression, we stained samples with anti-CD28-FITC and anti-CD40L-FITC, respectively, in order to observe the changes in the CD28 and CD40L levels of T cells in the different groups. Antibodies for flow cytometry were purchased from BioLegend. Stained cells were analysed using a BD-LSRFortessa flow cytometer (BD Biosciences). Data were analysed using the FlowJo software package.

### 3.7 Statistical analysis

Transcriptomic data were analysed using R version 4.2.2. Partial packages were employed to analyse the data; these processes are described in the data collection and processing sections. In addition, the packages dplyr, reshape2, and tidyverse were employed for data conversion and analysis, and ggplot2, ggpubr, ggstatsplot, pheatmap, RColorBrewer, and VennDiagram were used to visualise the results of data analysis. For comparisons, normally distributed data were analysed using the Student’s *t*-test or one-way analysis of variance (ANOVA), and the results are reported in the form mean ± standard deviation; P < 0.05 was considered to represent statistical significance. Statistical analyses of flow cytometry data were conducted using the GraphPad Prism software package (version 8.0; GraphPad Software, US).

## 4 Discussion

The inflammatory immune response to surgical injury can progress to a dysregulated state (20). Early intervention is essential to manage the systemic inflammatory state; therefore, predictive biomarkers for postoperative outcomes may positively affect outcomes. Many inflammatory mediators have been evaluated as potential biomarkers in patients with non-surgical sepsis, but only a small number of studies have focused on surgical patients, and even fewer have looked prospectively at blood samples taken early after surgery before clinical signs become evident. However, studies investigating the link between immunity and metabolism have provided new perspectives on the state of immune imbalance after surgery, thereby expanding our understanding of the immunological basis of postoperative complications, and identified prognostic biological signatures. In this study, we used plasma CRP levels to assess the extreme phenotypic state of PSID and conducted a comprehensive integrated analysis of immune- and metabolic-related gene expression in PSID. CRP level is a good indicator of infection status. CRP > 250 mg/L as PSID classification standard, the control for > 75mg/L. Postoperative CRP>150 mg/L (at 3–5 days postoperatively) is the most sensitive biochemical indicator of infection (21), and patients with CRP levels lower than 135mg/L on the 4th day after surgery are less likely to develop postoperative infectious complications (22). ROC analysis of cutoff points associated with AUC≥0.8 has identified relatively similar CRP levels (123 to 190 mg/l) as the optimal cutoff point for balancing sensitivity and specificity in the identification of surgical infection for various gastrointestinal cancers (23). Therefore, the criteria we selected are in good agreement with the critical values identified in the above studies.

We confirmed that immunity and metabolism were significantly and positively correlated during PSID, and that, at the PSID stage, the activation of myeloid cells and the suppression of lymphoid cells were significant. WGCNA hub genes were identified during PSID: these consisted of *CD28* and *CD40LG* downregulation and *MAPK14* and *S100A12* upregulation.

Inflammation is essential in reducing exposure to harmful cell debris and pathogens and in promoting healing. The immune response is balanced between the innate and adaptive immune systems through proinflammatory and anti-inflammatory processes (24). Disruption of this balance increases the risk of development of life-threatening inflammatory complications, including infections, systemic inflammatory response syndrome (SIRS), or sepsis. Myeloid-derived suppressor cells consist of immature myeloid cells, including progenitors or precursors of monocytes, neutrophils, and dendritic cells (25), and are characterised primarily by their inhibitory properties (to both innate and adaptive immunity) and their release in response to various inflammatory and/or infectious signals (26). The number of these cells is substantially increased in experimental models of sepsis (27–30). The development of severe lymphopenia in patients with sepsis is a major feature of adaptive immune sepsis (31). Retrospective studies have shown that persistent lymphatic disease is associated with an increased risk of death and nosocomial infections in patients with sepsis (32–34). Our results indicated that these characteristics are also present at the PSID stage; we also described the changes in the immune scores of myeloid and lymphoid cells, as well as in the genes enriched in these cells (**Figure 2** and **Supplementary Figure 2**).

Key proinflammatory responses during sepsis include activation of the complement system, coagulation system, vascular endothelium, neutrophils, and platelets, whereas immune suppression is primarily caused by the reprogramming of antigen-presenting cells, apoptosis, and exhaustion of lymphocytes (2). Genes enriched for specific immune-related KEGG pathways were plotted using cNetplots (**Supplementary Figure 1A**). The results showed that complement and coagulation cascades were upregulated.

The interplay between the complement system and coagulation has clinical implications in inflammatory pathogenesis, in which complement–coagulation interactions contribute to the development of life-threatening complications (16–18). In addition, genes associated with the neutrophil extracellular trap (NET) formation pathway were upregulated in the entire population. NET is a regulated form of neutrophil cell death that contributes to host defence against pathogens and is linked to various diseases (19, 20).

CD28 functions as a co-stimulator of T-cell receptor-mediated antigen activation, while the interleukin-7 receptor is critical in mediating T-cell maturation and survival (35, 36). Downregulation of *CD28* during inflammatory dysregulation is associated with outcomes following major trauma and sepsis (35, 37). The similarity between the downstream signalling pathways of the insulin receptor and CD28 suggests that CD28 may regulate glucose utilisation in a manner similar to that of the insulin receptor, coordinating the control of T cell activation and metabolism (38). In non-diabetic patients, levels > 10 mmol/L may remain elevated for days following surgery. Increased serum glucose concentration and peripheral insulin resistance result in persistently elevated blood glucose levels (39). CD28 co-stimulation of human peripheral blood T cells enhances the expression of glucose transporters, glucose uptake, and glycolysis (38, 40), which suggests that CD28 expression is of great significance for the prediction and diagnosis of PSID.

CD40 is a membrane glycoprotein belonging to the tumour necrosis family receptor superfamily, and its ligand CD40LG is a glycoprotein belonging to the tumour necrosis factor family. CD40–CD40LG interactions are essential in immune responses and inflammation (41–43). Dendritic cells (DCs) activate CD4+ T cells, which in turn provide help to B cells for antibody production (44–46). Importantly, CD40LG is transiently expressed in T cells and other non-immune cells under inflammatory conditions (45, 47). This finding suggests its importance in predicting inflammation in the early stages. Expression of CD40LG on various vascular cells contributes to the pathogenesis of atherosclerosis, thrombosis, and inflammatory processes (48, 49). Previous studies have shown that CD40LG is also closely associated with insulin resistance (50–52).

Interestingly, PSID-related hub genes encoding S100 proteins were upregulated. S100 proteins are potent TLR4 ligands with the potential to stimulate monocytes and to amplify ongoing inflammation (53–55) and myeloid expansion (56). The human *S100A12* gene, located on chromosome 1q21 (57), plays a role in the innate immune response and is associated with certain autoimmune responses. Human S100A12 is significantly overexpressed in the inflammatory compartment, and elevated serum levels of S100A12 are observed in patients with various inflammatory, neurodegenerative, metabolic, and neoplastic diseases (58, 59). This evidence suggests the strong potential of S100A12 as a sensitive and specific diagnostic marker for PSID.

MAPK14 plays a direct and essential role in relieving inhibitory control by autophagy (60). In our study, the upregulation of *MAPK14* observed in the postoperative hyper-inflammatory state suggests this suppressed state. MAPK14-driven metabolic reprogramming sustains the production of NADPH, an important cofactor for several reduction reactions, and the maintenance of a proper intracellular redox environment, thereby reducing the levels of reactive oxygen species (61).

In this study, we used co-expression network analysis to explore the changes in immune-related hub genes and metabolic hub genes occurring in PSID and identified differences in myeloid and lymphoid cells between PSID and Control groups. Our findings provide novel insights into the pathogenesis of PSID.

## Data availability statement

The original contributions presented in the study are included in the article/**Supplementary Material**. Further inquiries can be directed to the corresponding authors.

## Ethics statement

The animal study was approved by Standing Committee on Animals at the Tongji University. The study was conducted in accordance with the local legislation and institutional requirements.

## Author contributions

Project design and supervision: CL, HZ, and QL. Generation of critical concepts: SC and JT. Experimental work and data analysis: MF, QJ, MZ, and FM. Writing and revision of the manuscript: SC and JT.
