# Supplementary material for: Identification of potential hub genes linked to immune and metabolic alterations in postoperative systemic inflammatory dysregulation
Source: Front Immunol. 2023 Sep 8;14:1238774. doi: 10.3389/fimmu.2023.1238774 (PMC10515200; doi:10.3389/fimmu.2023.1238774)
Supplement: Supplementary file 1 [file DataSheet_1.pdf]

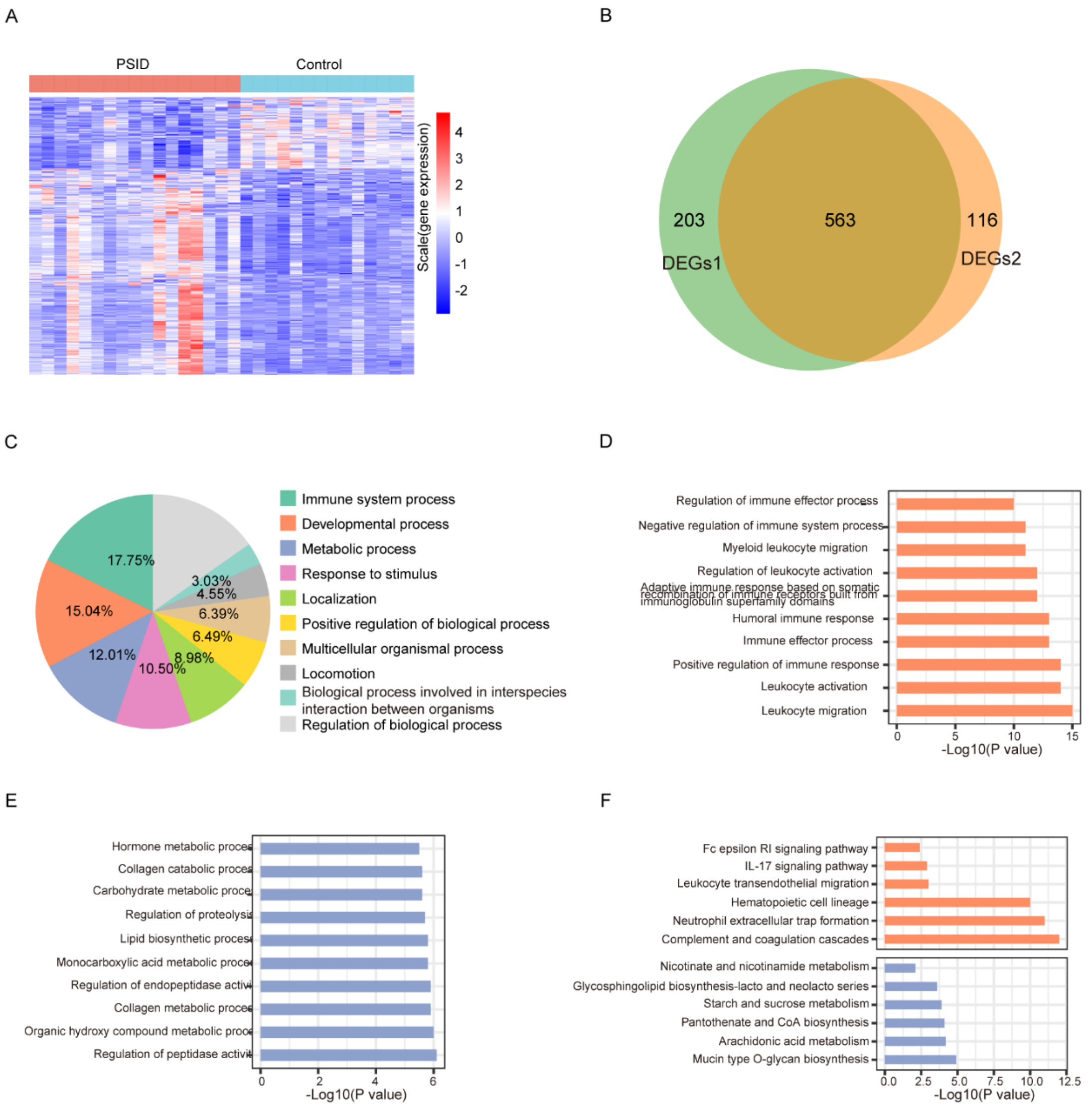

Supplementary Figure 1. Widespread immune system process and metabolic process changes are associated with PSID between the ages of 50 and 80. (A) Heatmap of 679 differentially expressed genes between the PSID and Control patients. The DEGs were filtered with |log2 fold change (FC)|≥1 and adjusted p value < 0.05. **(B)** Biological function of 679 DEGs biological processes annotated by Gene ontology (GO) enrichment analysis. **(C)** Top 10 biological processes associated with immunology and metabolism enriched by 679 DEGs with GO enrichment analysis. **(D-F)** Immune-related pathways and metabolism-related pathways were significantly enriched by 679 DEGs with KEGG enrichment analysis.

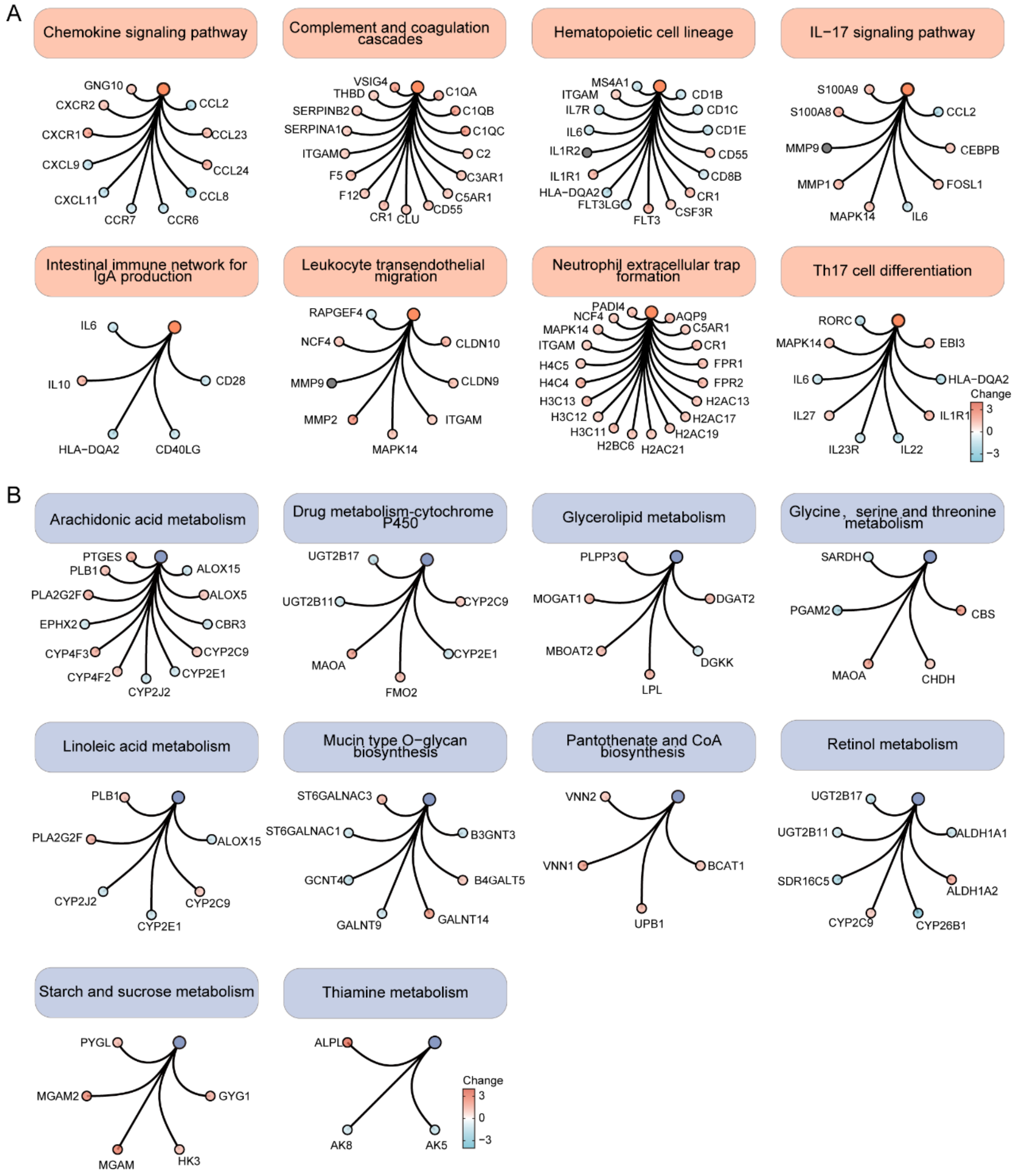

Supplementary Figure 2. Differentially expressed genes were enriched in each pathway. (A) Enrichment analysis of the DEGs in eight immune-related pathways. **(B)** Enrichment analysis of DEDs in ten metabolism-related pathways.

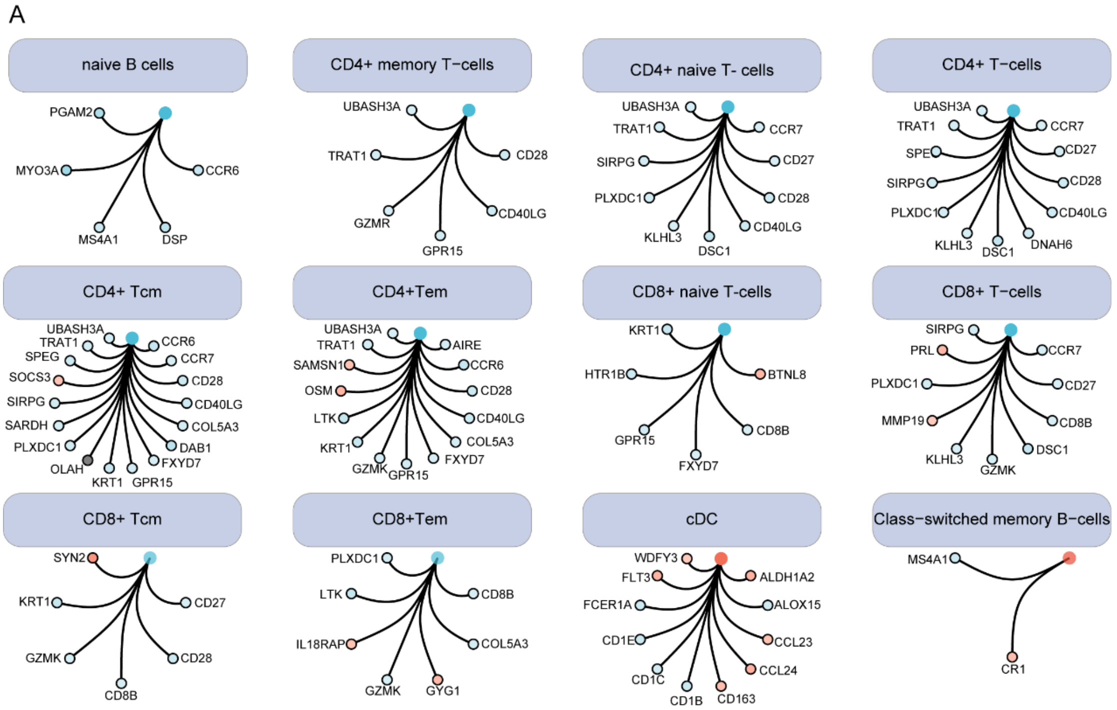

Supplementary Figure 3. The enriched DEGs for specific immune cells.

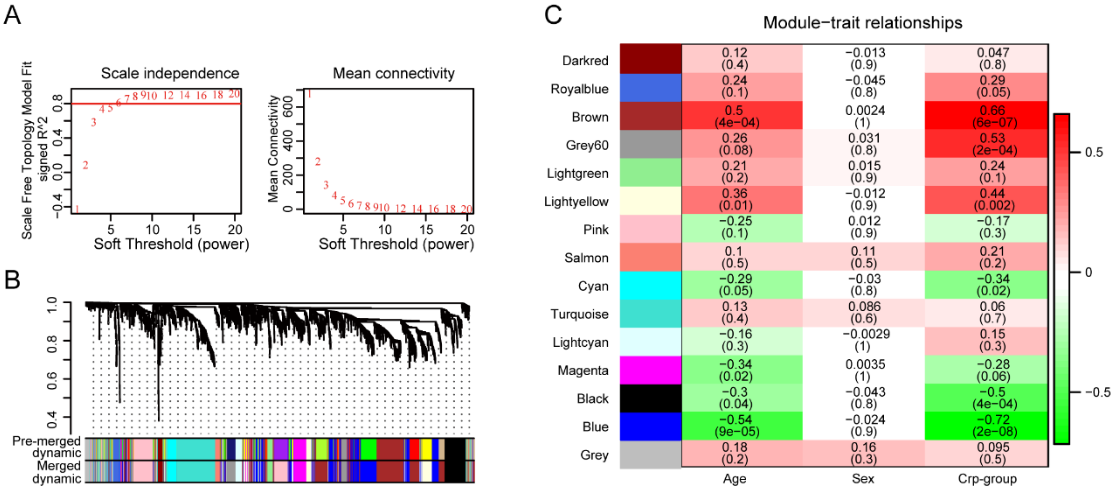

Supplementary Figure 4. Preprocessing of weighted gene co-expression network analysis. **(A)** Scale independence and mean connectivity of various soft threshold values (β). Red numbers indicate the different soft threshold values (1–20), while red lines indicate the selected cutoff values, with scale independence > 0.8. **(B)** Cluster dendrogram obtained from the transcriptomic data of the PSID in the database with average hierarchical linkage clustering. The coloured row underneath the dendrogram shows the module assignment determined by dynamic tree cut and merged dynamics. Altogether, 21 co-expression modules were constructed and are displayed in different colours. **(C)** Module-trait associations. Each row represents a module, while each column represents a trait. The number in the rectangle is the

correlation coefficient, and the number in brackets is the corresponding P value. The table was colour-coded using correlations based on the colour legend.

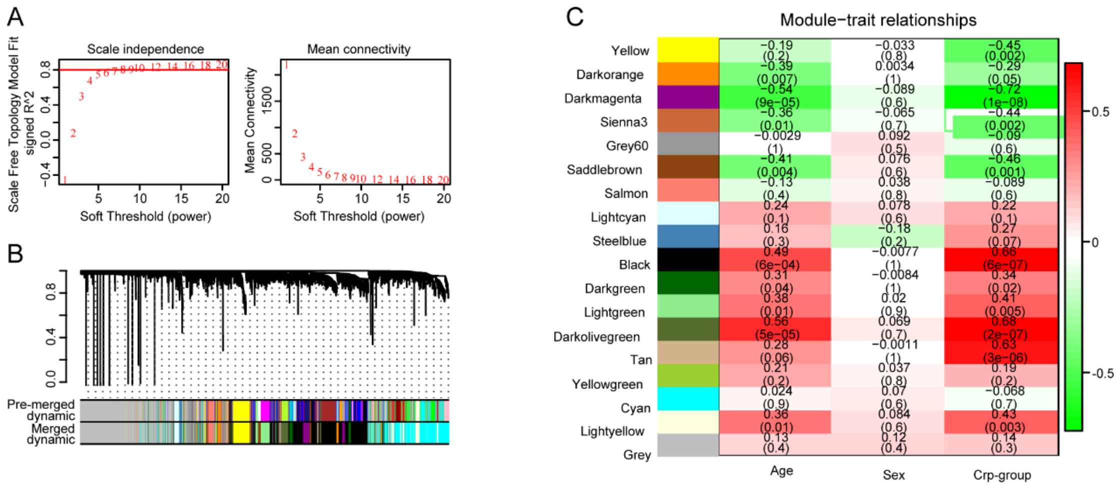

Supplementary Figure 5. Preprocessing of weighted gene co-expression network analysis. **(A)** Soft threshold and network connectivity. The networks of the PSID and Control groups formed a scale-free network in which the mean indicated node connectivity. **(B)** Clustering dendrograms of all metabolism-related genes with dissimilarities based on topological overlap, together with the assigned module colours. Altogether, 37 co-expression modules were constructed and are displayed in different colours. **(C)** Module-trait associations. The number in the rectangle is the correlation coefficient, and the number in brackets is the corresponding P value. The table was colour-coded using correlations based on the colour legend.
